# VX-770-mediated potentiation of numerous human CFTR disease mutants is influenced by phosphorylation level

**DOI:** 10.1038/s41598-019-49921-4

**Published:** 2019-09-17

**Authors:** Guiying Cui, Brandon B. Stauffer, Barry R. Imhoff, Andras Rab, Jeong S. Hong, Eric J. Sorscher, Nael A. McCarty

**Affiliations:** Division of Pulmonology, Allergy/Immunology, Cystic Fibrosis, and Sleep, Department of Pediatrics, Emory+Children’s Center for Cystic Fibrosis and Airways Disease Research, Emory University School of Medicine and Children’s Healthcare of Atlanta, Inc., 2015 Uppergate Drive, Atlanta, GA 30322 USA

**Keywords:** Permeation and transport, Physiology

## Abstract

VX-770 (ivacaftor) is approved for clinical use in CF patients bearing multiple CFTR mutations. VX-770 potentiated wildtype CFTR and several disease mutants expressed in oocytes in a manner modulated by PKA-mediated phosphorylation. Potentiation of some other mutants, including G551D-CFTR, was less dependent upon the level of phosphorylation, likely related to the severe gating defects in these mutants exhibited in part by a shift in PKA sensitivity to activation, possibly due to an electrostatic interaction of D551 with K1250. Phosphorylation-dependent potentiation of wildtype CFTR and other variants also was observed in epithelial cells. Hence, the efficacy of potentiators may be obscured by a ceiling effect when drug screening is performed under strongly phosphorylating conditions. These results should be considered in campaigns for CFTR potentiator discovery, and may enable the expansion of VX-770 to CF patients bearing ultra-orphan CFTR mutations.

## Introduction

Cystic fibrosis (CF), an inherited disease found mainly in the Caucasian population, is caused by mutations in the Cystic Fibrosis Transmembrane conductance Regulator (CFTR). Over 2,000 mutations in CFTR have been identified thus far, with over 300 well established as directly associated with disease (www.cftr2.org). CFTR is a member of the ATP-Binding Cassette (ABC) Transporter Superfamily but functions as a chloride channel and bears two nucleotide binding domains (NBDs) and twelve transmembrane helices (TMs). Unlike most other ABC proteins, CFTR also includes a regulatory (R) domain containing about 200 residues^1^, and encoding multiple consensus sites for phosphorylation by protein kinases A (PKA) and C (PKC). Experimental data and available homology models suggest that one CFTR protein as a monomer forms one chloride channel, which is verified by the recent Cryo-EM structures of zebrafish and human CFTR^2–5^.

CFTR channels are regulated by phosphorylation of the mostly unstructured R domain, together with interactions of MgATP at the two cytoplasmic NBDs. In total, human CFTR contains 11 PKA consensus sites that are sequentially phosphorylated and have distinct effects on channel gating^6^. Intracellular MgATP concentrations are in the millimolar range, which is more than ten times higher than the half maximal concentration of MgATP (about 50 µM) required for CFTR activation^1^. Therefore, phosphorylation is the primary mechanism for modifying CFTR activity *in vivo*. It previously has been reported that wildtype human CFTR (WT-CFTR), when activated by lower concentrations of PKA in the presence of MgATP, exhibits diminished open probability, shorter mean burst duration, and longer interburst durations compared to that of WT-CFTR channels activated at higher PKA concentration^6^. Evidence collected thus far indicates that the R domain undergoes structural rearrangement upon phosphorylation and exhibits dynamic, multisite interactions with NBD1, NBD2, and the C-terminus of CFTR that may underlie the graded response to PKA^7–9^. Taken together, R domain phosphorylation likely modulates CFTR channel activity through multiple conformational changes and numerous distinct mechanisms.

CFTR channels can be modulated by many small drug-like compounds, including the current FDA approved potentiator ivacaftor (VX-770), and correctors lumacaftor (VX-809) and tezacaftor (VX-661)^10–12^. VX-770 not only potentiates multiple variants in human CFTR, it also exhibits strong potentiation of murine CFTR and *Xenopus* CFTR^10,13,14^. NPPB, a well-known intracellular open pore blocker of human CFTR that blocks human CFTR regardless of channel activity level, was discovered to potentiate human CFTR in a phosphorylation-dependent manner; i.e., it only potentiated human CFTR when activated by a submaximal concentration of PKA^15^. Similar phenomena were also seen for potentiators P2 and P3; both potentiated human CFTR under conditions of lower phosphorylation^16^. Although the above compounds have provided proof-of-concept that CFTR protein function can be precisely modulated by small-molecule drugs, their exact working mechanism(s) and binding site (or sites) in human CFTR remain unclear.

Emerging data has verified the pleiotropic molecular defects caused by many CFTR mutations, and that efficacy of pharmacological monotherapy against single mutants is complex, as established for F508del- and N1303K-CFTR^11^. VX-770 has been approved by the FDA to treat patients with gating mutations including G551D and the ultra-orphan mutation P67L, and this now extends to 38 mutant CFTR variants^17^. The recent extension of FDA approval for available therapeutics to patients bearing ultra-orphan mutations has been based in part on *in vitro* evidence of activity. However, *in vitro* activity of drugs such as VX-770 against various disease-associated mutants of CFTR varies between laboratories^13,16,18,19^. Clarification of ways in which experimental conditions influence apparent efficacy of VX-770 may have important impact on assays used by industry and academia to identify new CFTR-targeted therapeutics. With this in mind, the present study was intended to determine whether efficacy of VX-770-mediated potentiation of CFTR is dependent upon channel activation state, and to probe more physiological and practical ways to characterize VX-770-mediated potentiation.

We previously found that VX-770 mildly potentiated WT-CFTR to similar levels when CFTR channels were activated by either 25.5 U/ml or 127.6 U/ml PKA in the presence of 1 mM MgATP. Similar results were obtained for P2 and P3; both potentiators failed to strongly potentiate WT- or D1152A-CFTR in the presence of 25.5 U/ml and 127.6 U/ml PKA. We later established that P3 could potentiate D1152A-CFTR when channels were activated in extremely low PKA (6.4 U/ml)^16^. Moreover, the channel blocker NPPB potentiated WT-CFTR when channels were activated under minimal PKA conditions^14–16^. Hence, the extent of CFTR channel activity controlled by PKA phosphorylation appears to play an essential role in modulating efficacy of potentiation, and can provide insight regarding the functional mechanism(s) that underlie CFTR potentiator compounds. However, the interaction between phosphorylation and potentiation has not been studied systematically to date.

## Methods

### Preparation of oocytes and cRNA

For electrophysiology experiments, Human CFTR cRNAs used in this study were prepared from constructs encoding the WT-CFTR gene in the pGEMHE vector (We thank the late Dr. David C. Gadsby for the donation of the WT-CFTR/pGEMHE construct). All mutants of CFTR were generated using the Quikchange site-directed mutagenesis kit (Stratagene, La Jolla, CA). The entire open reading frames of all mutant CFTR constructs were screened as previously reported^20^. *Xenopus laevis* oocytes were injected with 0.5–10 ng of CFTR cRNAs based on the experiment purpose, and were incubated at 17 °C in modified Liebovitz’s L-15 media. Modified L-15 media was further optimized by the addition of HEPEs (pH 7.5), penicillin, and streptomycin. Electrophysiology experiments were performed 24–96 hours following injection of cRNAs. Protocols for *Xenopus laevis* frog handling and oocyte collection were based on and adhere to NIH guidelines, and have been approved by the Institutional Animal Care and Use Committee of Emory University^20^.

### FRT cell line and primary cells

Fischer rat thyroid (FRT) cells with stable WT or variant CFTR expression were established by the Sorscher lab^21^. Cells were maintained in Coon’s modified Ham’s F12 medium, supplemented with 5% fetal bovine serum, and grown at 37 °C in a humidified incubator with 95% O_2_/5% CO_2_. Plating was at 1 × 10^6^ cells per well on Transwell permeable supports according to experimental requirements. Primary human bronchial epithelial cells (HBE) were obtained already seeded on Transwell permeable support from the Experimental Models Support Core, CF@LANTA RDP Center at Emory University. HBE cells were maintained at an air/liquid interface and cultured as described^22^.

### Electrophysiology

All electrophysiology pipettes used for inside-out macropatch and single-channel recordings were pulled from borosilicate glass (Sutter Instrument Co., Novato, CA) and pipette resistances ranged from 1–2 MΩ for macropatch and ~10 MΩ for single channel recording after filling with chloride-containing pipette solution (in mM): 150 NMDG-Cl, 5 MgCl_2_, 10 TES (pH 7.5), as previously reported^14,16^. Oocytes were shrunk in hypertonic solution followed by manual removal of the vitelline membrane before electrophysiology experiments. Inside-out patches were excised from CFTR-expressing oocytes, and CFTR channels were activated in cytoplasmic solution containing (in mM): 150 NMDG-Cl, 1.1 MgCl_2_, 2 Tris-EGTA, 10 TES, 1 MgATP (Adenosine 5’-triphosphate magnesium), and distinct concentrations of PKA (pH 7.45). All electrophysiology experiments were done at room temperature (22–23 °C). All macropatch recordings utilized an Axopatch 200B amplifier operated by pClamp 8.2 software; data were filtered at 100 Hz with a four-pole Bessel filter and acquired at 2 kHz. The following voltage protocol used in this project, as previously reported, was applied every 5 s: hold at Vm = 0 mV, followed by step to + 100 mV for 50 ms, then succeeded by a ramp down to −100 mV over 300 ms, before finally returning to 0 mV^16^. Single channel recordings were obtained using an Axopatch 200B amplifier and recorded to digital tape. Traces were played back with Clampfit 9.0. Both macropatch and single channel data were analyzed with Clampfit 10.2 software^20^.

### Ussing chamber analysis

CFTR-mediated transepithelial short-circuit Cl^−^ current (I_sc_) was measured under voltage clamp conditions (clamping voltage 0 mV) with an Ussing chamber system including a VCC-MC6 amplifier and EM-RSYS-2 chambers, using P2302T sliders, and P2020-S electrodes (Physiologic Instruments)^22^. Electrodes were prepared by adding about 1 cm of 3 M KCl/3% agar to the electrode tips according to the manufacturer’s protocol. The voltage offset and fluid resistance compensation were adjusted according to the manufacturer’s instructions using a blank filter. Filters were inserted into the chamber and the following solutions added (mM): basolateral solution composed of 115 NaCl, 5 KCl, 1 MgCl_2_, 2 CaCl_2_, 10 glucose, 10 HEPES, 25 NaHCO_3_ (pH 7.2); apical solution composed of 115 NaGluconate, 5 KCl, 1 MgCl_2_, 4 CaCl_2_, 10 glucose, 10 HEPES, 25 NaHCO_3_ (pH, 7.2). Both solutions were bubbled with a 95%/5% mixture of O_2_/CO_2_ and heated to 37 °C. FRT cells were activated by different concentrations of the CFTR agonist forskolin (FSK), followed by the potentiator VX-770 (1 µM). Amiloride (100 µM) was applied at the beginning of each experiment to block residual Na^+^ current in HBE cells. INH_172_ (CFTR_inh_172) was administered to the apical solution at the completion of each experiment to block CFTR-dependent I_sc_. Data were acquired using the Acquire and Analyze software and exported to Excel for analysis.

### Source of reagents

Unless otherwise noted, all reagents were obtained from Sigma Chemical Co. (St. Louis, MO). L-15 medium was obtained from Gibco/BRL (Gaithersburg, MD). CFTR_inh_172 was purchased from Calbiochem (Burlington, MA) and made as 50 mM stock in DMSO for future use. PKA was used at, or diluted from, 127.6 U/ml to indicated final concentrations for patch experiments (Promega, Madison, WI). VX-770 was initially made as a 10 mM stock solution dissolved in DMSO (Selleckchem, Houston, TX). P2 was kindly provided by Cystic Fibrosis Foundation Therapeutics. All chemicals used in this study were diluted to their final concentration in the relevant recording solution immediately prior to use^16^.

### Statistical analysis

Unless otherwise stated, data are presented as mean ± SEM as previously reported^16^. Paired or unpaired t-tests were performed using Sigmaplot 12.3 (San Jose, CA) for statistical analysis. In addition, we performed one-way ANOVA with the Bonferroni correction for multiple comparisons using Prism (La Jolla, CA). *P* < 0.05 was considered significantly different. *P < 0.05; **P < 0.01; ***P < 0.001^16^.

## Results

### CFTR activity is regulated by PKA-mediated phosphorylation

**Figure 1:**
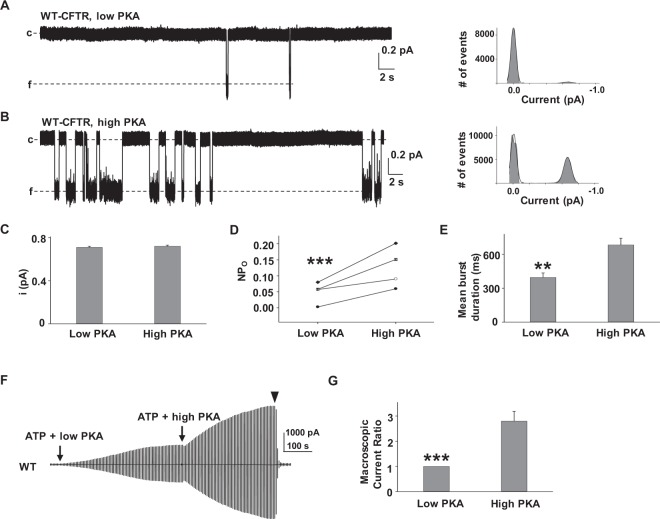
Single channel behavior in WT-CFTR is different in high and low PKA concentration. Representative single-channel current traces are shown for WT-CFTR in low PKA (**A**, 6.4 U/ml) and high PKA (**B**, 127.6 U/ml), from one inside-out membrane patch excised from *Xenopus* oocytes, with symmetrical 150 mM Cl^−^ solution in the presence of 1 mM MgATP. All traces were recorded at Vm = -100 mV. c = closed state; f = full open state. All-points amplitude histograms are shown in the right panels, where solid lines are fit results to a Gaussian function. Single channel amplitude (**C**), open probability (*n* = 4) (**D**), and mean burst duration (**E**) of WT-CFTR in low and high PKA conditions are shown. *n* = 5. (**F**) Representative macropatch currents of WT-CFTR recorded in inside-out mode with symmetrical 150 mM Cl^−^ solution under the following experimental conditions: channels were fully activated in 1 mM MgATP + 6.4 U/ml PKA followed by 1 mM MgATP + 127.6 U/ml PKA. A voltage-ramp protocol described in the Methods section was applied every 5 s. ▼, 10 µM CFTR_inh_172.

To determine the effects of PKA concentration in modulating WT-CFTR activity, we selected two PKA concentrations, 6.4 U/ml and 127.6 U/ml, for the following reasons: first, our previous findings showed that P2 and P3 distinctly potentiated WT- and D1152A-CFTR when activated in 6.4 U/ml PKA but not 127.6 U/ml; second, macroscopic current of WT-CFTR was very low (and highly variable) at PKA concentrations below 6.4 U/ml^16^. We maintained 1 mM MgATP throughout the whole project because it is the physiologically relevant concentration of ATP. We first tested single channel behavior of WT-CFTR expressed in *Xenopus* oocytes. A representative single channel trace recorded from one patch and the associated all-points amplitude histogram are shown for WT-CFTR activated by 6.4 U/ml PKA (Fig. 1A) and 127.6 U/ml PKA (Fig. 1B). Single channel amplitude was similar in both 6.4 U/ml and 127.6 U/ml PKA conditions (Fig. 1C), while open probability (Fig. 1D) and mean burst duration (Fig. 1E) were significantly lower and shorter with 6.4 U/ml PKA compared to 127.6 U/ml PKA. We then tested activation of WT-CFTR in bath solutions containing 6.4 U/ml and 127.6 U/ml PKA in excised inside-out macropatches (representative current trace is shown in Fig. 1F). After WT-CFTR current reached plateau using 6.4 U/ml PKA (~ 10 minutes post-exposure to PKA and ATP), CFTR current was further increased by exposure to high PKA (127.6 U/ml). Macroscopic current ratio, calculated as I_(maximum current in high PKA)_/I_(maximum current in low PKA)_, was 2.8 ± 0.39 (*n* = 9). The data suggest that WT-CFTR is activated by PKA in a concentration-dependent manner, even when current is allowed to reach plateau at each level of PKA evaluated; the difference between low and high PKA conditions likely reflects distinct sensitivities of the 11 consensus PKA phosphorylation sites in CFTR. In addition, the macroscopic currents of CFTR, which increased from low to high PKA here, may be affected not only by single channel amplitude (no change) and channel activity (open probability, increase), but also influenced by an increase in the total number of active channels (the total number of CFTR channel proteins per patch likely remained unchanged in this system).

**Figure 2:**
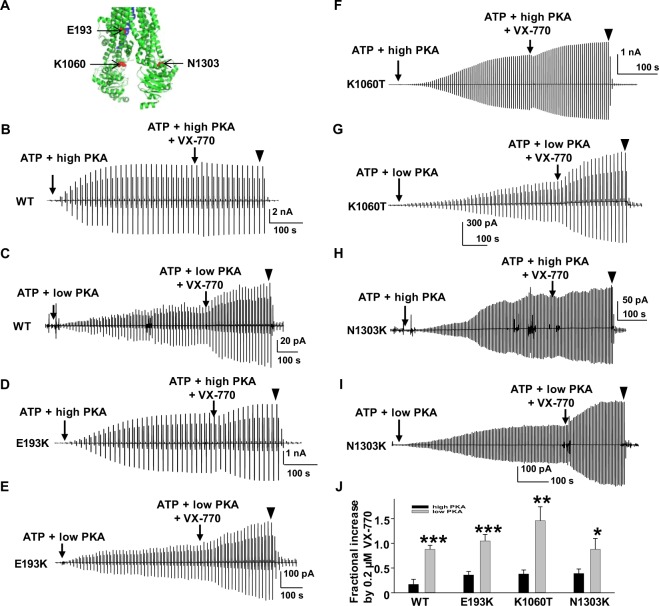
Cytoplasmic VX-770 potentiated WT-, E193K-, K1060T-, and N1303K-CFTR in a manner sensitive to phosphorylation-level. (**A**) Three amino acids E193, K1060, N1303 are shown as red spheres in the closed state human CFTR Cryo-EM model adopted from previous publication and prepared with PyMOL^5^. TM6 is indicated in blue shades. Representative macropatch currents of CFTR (WT: (**B**,**C**); E193K: (**D**,**E**); K1060T: (**F**,**G**); N1303K: (**H**,**I**)) were recorded in inside-out mode with symmetrical 150 mM Cl^−^ solution under the following experimental conditions: channels were activated in 1 mM MgATP + 6.4 U/ml (low PKA) or 127.6 U/ml PKA (high PKA) for ten minutes followed with addition of 0.2 µM VX-770 in the continuing presence of MgATP + PKA for about three minutes; currents then were blocked by 10 µM CFTR_inh_172 (▼). A voltage-ramp protocol described in the Methods section was applied every 5 s. **J**. Summary data for fractional increase of WT- and three disease CFTR mutants by VX-770 under each set of conditions are shown (Fractional increase = I_(ATP+PKA+VX-770)_/I_(ATP+PKA)_ − 1). Black bars are from experiments with high PKA and gray bars are from experiments with low PKA. WT: *n* = 6 for high PKA; *n* = 5 for low PKA. E193K, *n* = 5 for both high and low PKA. N1303K: *n* = 5 for high PKA; *n* = 4 for low PKA. K1060T, *n* = 5 for high PKA; *n* = 8 for low PKA. *P < 0.05, **P < 0.01 and ***P < 0.001 compared to high PKA condition.

Based upon our prior data, we hypothesized that efficacy of VX-770-mediated potentiation also would depend upon the level of phosphorylation^14,16^. To test this, we asked whether VX-770 potentiated WT-CFTR more robustly in 6.4 U/ml PKA compared to 127.6 U/ml PKA using excised inside-out macropatches. Representative current traces are shown in Fig. 2. CFTR channels were fully activated (~10 minutes post-exposure to PKA and MgATP), followed by the addition of 0.2 µM VX-770 in the continuing presence of MgATP and low or high PKA until current reached a plateau (approximately 3 minutes)^14,23^. CFTR channels were then inhibited by 10 µM INH_172_. The degree of potentiation was related to the concentration of PKA (0.17 ± 0.10, *n* = 6 for high PKA; 0.88 ± 0.08, *n* = 5 for low PKA) (Fig. 2J). The results verified our working hypothesis that VX-770 would potentiate WT-CFTR in a PKA concentration-dependent manner possibly by increase channel open probability and the number of active channels.

### VX-770 potentiated CFTR disease mutants dependent upon the level of phosphorylation

Given previous evidence together with the above data, we expanded our hypothesis to include not only wildtype, but also disease-causing mutations in CFTR, to determine whether they, too, would be potentiated by VX-770 in a phosphorylation-dependent manner^16^. We selected 6 variants based on the following criteria: (1) the most common mutations found in CF (including F508del (86.4%), G551D (4.4%), and N1303K (2.4%) (https://www.cff.org)); (2) Mutations located in different domains across the CFTR protein, including E193K of helix TM3 and K1060T of intracellular loop 4 (ICL); (3) Based on a report that the efficacy of potentiation by VX-770 at over 50 disease-associated mutations varied dramatically under the same experimental conditions^24^, we selected examples with very high (E193K), moderate (P67L), or low (F508del) potentiation by VX-770; (4) Different classes of CFTR mutations. For example, E193K and G551D (Class III) which cause a severe loss of ion conduction due to reduced open probability^25,26^, K1060T (Class III/IV) which is proposed to break a putative electrostatic interaction between K1060 and E267, causing mild CF disease^27,28^, N1303K and F508del (Class II) mutations with severe trafficking abnormalities^11,24^, and P67L, primarily a Class II trafficking defect mutation that recently has been recognized to also cause a gating problem^21,29^.

Figure 2A shows 3 amino acid positions E193 (TM3), K1060 (ICL4), and N1303 (NBD2) in the human CFTR Cryo-EM structure adopted from Chen *et al*.^5^. We examined effects of 0.2 µM VX-770 on these variants using the same protocol as for WT-CFTR shown in Fig. 2B,C. Representative current traces and summary data are shown. Similar to WT-CFTR, VX-770-mediated potentiation of E193K-, K1060T-, and N1303K-CFTR was marginal when channels were activated by high concentrations of PKA (127.6 U/ml), with a fractional increase of approximately 30%. In contrast, the same concentration of VX-770 potentiated the three variants significantly more robustly (approximately 100% increase) when pre-activation was mediated by low PKA (6.4 U/ml). These data provide strong evidence to support our hypothesis that VX-770 potentiates wildtype as well as disease-associated mutants in a graded phosphorylation-dependent manner. The phenomenon is not unique to VX-770 as potentiator, since P2 also activated WT-, E193K-, K1060T-, and N1303K-CFTR in a fashion that depended on the level of PKA (Supplementary Fig. 1)^16^.

### Potentiation by VX-770 in disease-associated mutants exhibits distinct responsiveness to PKA

**Figure 3:**
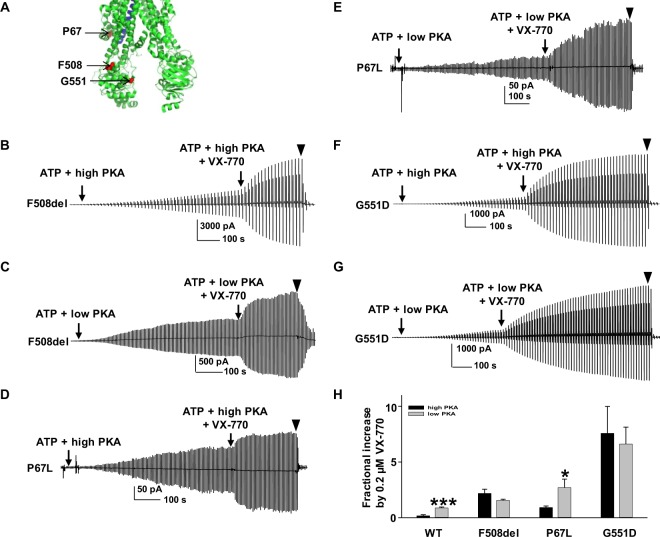
VX-770 potentiated F508del-, P67L-, and G551D-CFTR in a manner less sensitive to phosphorylation level. (**A**) Three amino acids F508, P67, and G551 are shown as red spheres in the closed state human CFTR Cryo-EM model adopted from previous publication and prepared with PyMOL^5^. TM6 is indicated in blue shades. Representative macropatch currents of CFTR (F508del: (**B**,**C**); P67L: (**D**,**E**); G551D: (**F**,**G**)) recorded in inside-out mode with the same experimental conditions as Fig. 2. ▼, 10 µM CFTR_inh_172. (**H**) Summary data for fractional increase of WT- and three disease mutant CFTR variants by VX-770 under high and low PKA are shown. F508del: *n* = 10 for high PKA; *n* = 5 for low PKA. P67L: *n* = 6 for high PKA; *n* = 5 for low PKA. G551D: *n* = 5 for high PKA; *n* = 6 for low PKA. *P < 0.05 and ***P < 0.001 compared to high PKA condition.

We further determined the efficacy of VX-770-mediated potentiation for P67L-, F508del-, and G551D-CFTR. Representative current traces and summary data are shown in Fig. 3. The positions of P67 (Lasso motif), F508 (NBD1), and G551 (NBD1) are labeled as red spheres in the Cryo-EM human CFTR structure shown in Fig. 3A. We tested effects of VX-770 on the above variants using the same protocol as for WT-CFTR, i.e., 10 minute exposure to 1 mM MgATP plus low or high concentrations of PKA, followed by addition of 0.2 µM VX-770 in the presence of PKA and MgATP to allow currents to reach a plateau. P67L-CFTR was potentiated with phosphorylation-level dependence similar to WT-CFTR but much more effectively. Surprisingly, F508del-CFTR was comparably potentiated by 0.2 µM VX-770 at both 6.4 U/ml and 127.6 U/ml PKA, although efficacy of potentiation was much higher than that seen for WT-CFTR (1.62 ± 0.51, *n* = 5 for 6.4 U/ml PKA; 2.18 ± 0.38, *n* = 10 for 127.6 U/ml PKA). Hence, potentiation of F508del-CFTR by VX-770 occurred in a manner independent of PKA dosing under these conditions. Interestingly, potentiation of G551D-CFTR was apparently insensitive to phosphorylation level, although the degree of potentiation was extremely high (>6-fold under both conditions). These results suggest two questions: (1) why does the sensitivity to VX-770-mediated potentiation differ for F508del- and G551D-CFTR compared to other CFTR variants, in terms of dependence upon phosphorylation level?; and, (2) what are possible mechanisms that intensify VX-770-mediated potentiation of F508del-, P67L-, and G551D- relative to WT-CFTR? We performed the following experiments to address these questions.

### Apparent phosphorylation-level insensitive potentiation by VX-770 in F508del-CFTR and impact on PKA sensitivity

**Figure 4:**
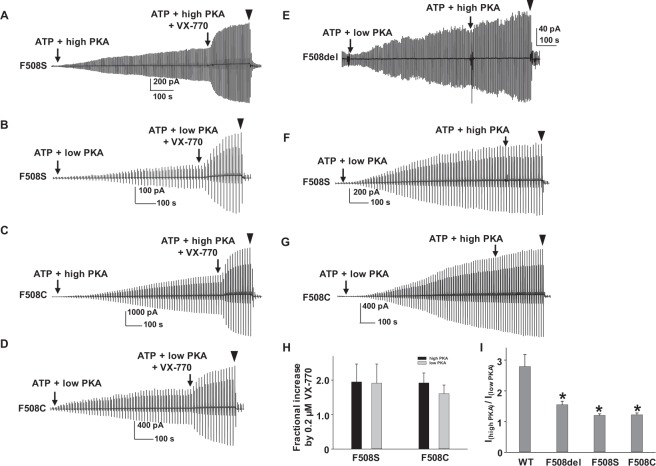
VX-770 potentiated F508S- and F508C-CFTR similarly to F508del-CFTR. VX-770 (0.2 µM) potentiated F508S-CFTR in high (**A**) and low (**B**) PKA concentrations with 1 mM MgATP. Representative current traces of F508C-CFTR potentiated by VX-770 in high (**C**) and low PKA (**D**) conditions also are shown. ▼, 10 µM CFTR_inh_172. Summary data for F508S- and F508C-CFTR potentiated by VX-770 are shown in (**H**). F508C: *n* = 7 for high PKA; *n* = 6 for low PKA. F508S: *n* = 6 for high PKA; *n* = 4 for low PKA. Representative current traces of F508del- (**E**), F508S- (**F**), and F508C- (**G**) CFTR activated by low PKA then by high PKA in the presence of 1 mM MgATP are shown. Summary data for the ratio of three F508 mutants in high and low PKA conditions are shown in panel **I** and calculated based on the equation in Fig. 1. *n* = 6 for F508del; *n* = 6 for F508S; *n* = 4 for F508C. *P < 0.05 compared to WT.

While F508del causes severe CF, two other mutations at F508 (F508S and F508C) are known to confer mild clinical phenotype. Although F508del has been recognized to cause multiple defects including mistrafficking in mammalian cells, channel gating defects, and instability at the epithelial plasma membrane following rescue by low temperature or pharmacological correction, F508C-CFTR is permissive for maturation with only a minor gating defect^11,30–33^. We evaluated whether F508S- and F508C-CFTR are potentiated by VX-770 in a manner similar to F508del-CFTR (i.e. with apparent phosphorylation-level insensitivity). Representative current traces and summary data are shown in Fig. 4. Like F508del-CFTR, both F508S- and F508C-CFTR were potentiated by VX-770 to a similar degree regardless of whether the channels were activated by low or high PKA. These data suggest that the apparent insensitivity of VX-770-mediated potentiation of F508del-CFTR under these conditions is not unique to this variant. We also tested whether F508del-, F508S-, or F508C-CFTR exhibited PKA concentration dependent activation different from that of WT-CFTR. We tested the three variants according to the protocol shown in Fig. 1; representative current traces are shown in Fig. 4E–G. As summarized in Fig. 4I, the three F508 mutants had significantly altered PKA sensitivity compared to WT-CFTR: high PKA (127.6 U/ml) only slightly increased these mutant CFTR currents following activation by low PKA (6.4 U/ml). We conclude that the unexpected behavior of these three mutants with respect to VX-770-mediated potentiation may be attributable to a PKA-mediated effect, which is already saturated at 6.4 U/ml PKA. The data also suggest that the F508del-CFTR gating defect at least partially results from an alteration in PKA sensitivity.

### P67L exhibits enhanced sensitivity to VX-770 potentiation

**Figure 5:**
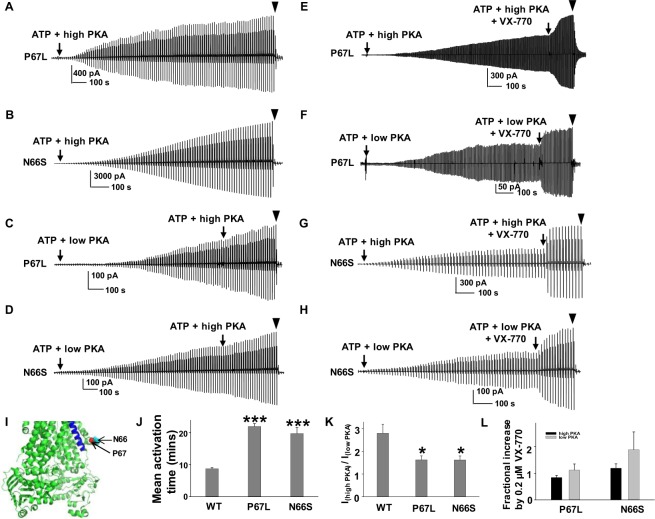
Activation rates of P67L- and N66S-CFTR are lower compared to WT-CFTR under high PKA conditions. Both P67 and N66 are located in the Lasso motif shown in panel **I** as red spheres in the closed state human CFTR Cryo-EM model prepared with PyMOL^5^. TM6 is indicated in blue color. (**A**,**B**) P67L- and N66S- activation durations are significantly longer than WT-CFTR in 1 mM MgATP + 127.6 U/ml PKA condition. (**C**,**D**) Both P67L- and N66S-CFTR exhibited a shift in PKA sensitivity. (**E**,**F**) Representative current traces of P67L potentiated by VX-770 after channels were fully activated in both high and low PKA conditions. (**G**,**H**) Representative current traces of N66S potentiated by VX-770 after channels were fully activated in both high and low PKA condition. ▼, 10 µM CFTR_inh_172. Summary data for activation durations are shown in panel (J). *n* = 30 for WT, *n* = 9 for P67L, and *n* = 10 for N66S. ***P < 0.001 compared to WT. Summary data for the ratio of P67L- and N66S-CFTR currents in low and high concentration of PKA are shown in panel (K) calculated based on the equation in Fig. 1. *P < 0.05 compared to WT. Summary data of P67L- and N66S-CFTR potentiated by VX-770 are shown in panel (L). *n* = 6 for both P67L and N66S in both low and high PKA conditions.

P67 is located immediately adjacent to Lasso helix 2 (Fig. 5)^5^. N66 is also situated in the Lasso domain and N66S has been associated with mild disease. P67L-CFTR exhibits trafficking and gating defects with normal single channel conductance, although the mechanism responsible for the gating defect remains unknown^21^. We hypothesized that P67L-CFTR channel gating might impact efficacy of VX-770 potentiation in a manner similar to that observed for F508del-CFTR. Surprisingly, we found that both P67L- and N66S-CFTR activated much more slowly than WT-CFTR under the same experimental conditions following treatment with 127.6 U/ml PKA and 1 mM MgATP. We defined activation duration as the time required to reach a plateau in the presence of high PKA. Representative current traces for P67L- and N66S-CFTR, together with summary data, are shown in Fig. 5. In addition, both P67L- and N66S-CFTR have significantly altered sensitivity to PKA, behaving more like F508 mutants than like WT-CFTR (Fig. 5K,L). These findings suggest that in our previous experiment (Fig. 3), P67L-CFTR was potentiated by VX-770 prior to channels becoming fully activated, since the channels were exposed to PKA and ATP for only 10 minutes. We therefore retested potentiation of P67L- and N66S-CFTR by VX-770 with a new protocol in which channels were allowed to fully activate (~20 minutes incubation) prior to addition of 0.2 µM VX-770 in the presence of ATP and PKA (Fig. 5). The fractional increase by 0.2 µM VX-770 for both P67L- and N66S-CFTR was reduced when channels were fully activated (~20 minutes incubation) in comparison to conditions leading to partial activation (~10 minute incubation). Furthermore, both mutants exhibited potentiation seemingly insensitive to phosphorylation level when fully activated. Taken together, we conclude that the P67L gating defect is directly related to altered PKA sensitivity, a finding that supports previous reports that the R domain is possibly involved in CFTR gating regardless of its phosphorylation state and may interact with the Lasso motif^3,5^.

### G551D-CFTR exhibits strongest potentiation by VX-770 among mutants tested here

**Figure 6:**
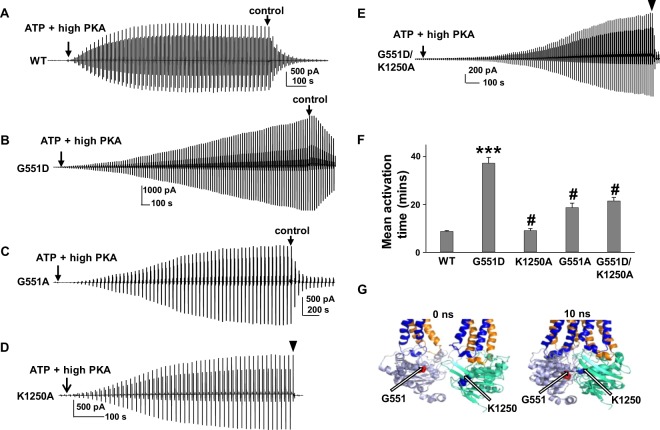
Activation of G551D-CFTR is slowed compared to WT-CFTR under the same experimental conditions. Representative current traces of WT- (**A**) and G551D-CFTR (**B**) recorded in symmetrical 150 mM Cl^−^ solution with 1 mM MgATP + 127.6 U/ml PKA using excised inside-out macropatch. The recordings were arbitrarily halted at 52 minutes even if the channels were not fully activated. (**C**) G551A-, (**D**) K1250A-, and (**E**) G551D/K1250A-CFTR activation durations were determined under the same experimental conditions as (**A**,**B**). Control, intracellular solution alone, without ATP or PKA. ▼, 10 µM CFTR_inh_172. Summary data are shown in panel (**F**). The estimated activation duration of G551D-CFTR is significantly longer than that of WT-CFTR (***P < 0.001 compared to WT). The activation duration of K1250A-CFTR is similar to that of WT-CFTR. Activation durations of G551A-, K1250A-, and G551D/K1250A-CFTR were significantly shorter compared to G551D-CFTR (#*P* < 0.001 compared to G551D-CFTR) (**F**). *n* = 30 each for WT-, G551D-, and G551A-CFTR. *n* = 13 for K1250A-CFTR. *n* = 18 for G551D/K1250A-CFTR. (**G**). Amino acid G551 is shown as red spheres and K1250 is shown as blue spheres in the closed state (0 ns) and open state (10 ns) simulations of the CFTR homology model adopted from our previous publication^39^ and prepared with PyMOL^5^. TM1–6 are indicated in blue shades and TM7–12 are indicated in orange shades. NBD1 is indicated in light blue and NBD2 is indicated in light green.

Unlike WT-CFTR, G551D-CFTR was potentiated over 6-fold by 0.2 µM VX-770 at both low and high PKA concentrations (Fig. 3). G551D is a representative Class III mutation with severe gating defects that include very low open probability and brief burst duration^3,34–36^. We hypothesized that the extreme sensitivity of G551D-CFTR to potentiation may reflect an as yet undiscovered mechanism. Dawson and colleagues reported that activation of G551D-CFTR by forskolin and IBMX in whole oocytes took significantly longer compared to WT-CFTR. In the same studies, deactivation of G551D-CFTR was slower following wash-out of forskolin and IBMX compared to WT-CFTR^37^. We determined the activation duration of G551D-CFTR in excised inside-out macropatches pulled from oocytes. As shown in Fig. 6, WT-CFTR required approximately 10 minutes (8.75 ± 0.35, n = 30) to be fully activated by 1 mM MgATP + 127.6 U/ml PKA^23^, while G551D-CFTR frequently was still not fully activated after 52 minute exposure to 1 mM MgATP + 127.6 U/ml PKA (recording was halted after 52 minutes to avoid disruption of the patch). These results are consistent with an earlier report^37^. With estimated mean activation duration for G551D-CFTR calculated in this manner was 37.23 ± 2.41 minutes (n = 30) (Fig. 6F).

Unlike G551D, which causes severe disease, G551S causes mild CF disease. Unfortunately, the complete mechanism behind this effect remains unclear. Hwang and Sheppard previously proposed that the negatively charged aspartic acid replacing the glycine at position 551 may disrupt a hydrogen bond formed between the γ-phosphate of ATP and the main chain hydrogen in glycine, blocking the subsequent signal transmission required for channel opening^38^. The same sort of abnormality could occur in G551S. We functionally tested the G551S mutation, but surprisingly, could not recover CFTR channels in oocytes even after multiple experiments. We therefore established the G551A mutation and found activation duration of the mutant was significantly longer than WT-, but distinctly shorter than G551D-CFTR (Fig. 6). We previously predicted based on molecular dynamics simulations using a CFTR homology model that D in G551 moved close to lysine at position 1250 during NBD dimerization, and might establish an aberrant electrostatic interaction (Fig. 6G)^39^. We hypothesized that the extremely slow activation of G551D-CFTR may be due to an electrostatic interaction of this sort, whereas G551A-CFTR would exhibit shortened activation duration due to absence of this bond. To test this, we studied the K1250A single mutant as well as the G551D/K1250A double mutant; in both cases the D551-K1250 electrostatic interaction should be absent. Representative current traces showing K1250A- and G551D/K1250A-CFTR together with summary data are shown in Fig. 6. The estimated activation duration of K1250A-CFTR is similar to that of WT-CFTR, while activation duration for G551D/K1250A-CFTR channels were similar to that of G551A-CFTR, significantly longer than that of WT-CFTR, but distinctly shorter than that of G551D-CFTR (Fig. 6F). This comparison, together with the much slower deactivation rates in G551D- but not G551A-CFTR upon washout of ATP (compare Fig. 6B to Fig. 6C, and see further below), strongly supports the idea that D551 forms an electrostatic interaction with K at the 1250 position, impeding G551D channel gating. The above findings also suggest that experiments using G551D-CFTR shown in Fig. 3 were undertaken before channels were fully activated, since we only exposed G551D-CFTR to 1 mM MgATP + high or low PKA for ~10 minutes prior addition of VX-770.

**Figure 7:**
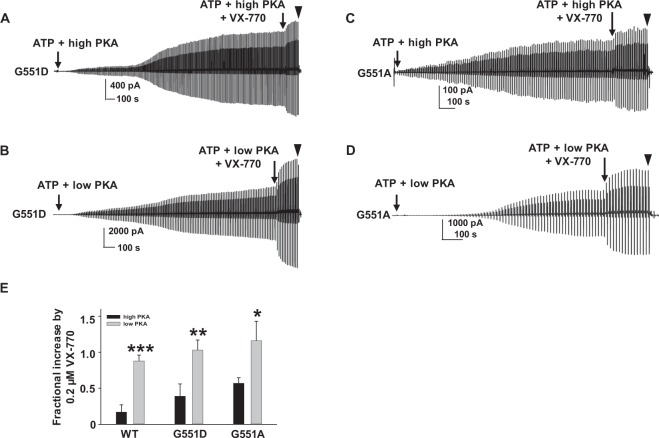
Cytoplasmic VX-770 potentiates G551D- and G551A-CFTR in a phosphorylation-dependent manner when channels were fully activated, albeit with much lower efficacy. Representative current traces of G551D- (**A**,**B**) and G551A-CFTR (**C**,**D**) recorded under the same conditions as in Fig. 3, except that channels were fully activated in 1 mM MgATP plus low or high PKA before addition of VX-770 in the continuing presence of ATP and PKA. ▼, 10 µM CFTR_inh_172. Summary data for effects of 0.2 µM VX-770 on WT-, G551D-, and G551A-CFTR are shown in (**E**). *P < 0.05, **P < 0.01, and ***P < 0.001 compared to high PKA. G551D: *n* = 8 for high PKA; *n* = 7 for low PKA. G551A: n = 5 for high PKA; *n* = 6 for low PKA. Data for WT-CFTR are the same as in Fig. 2.

In order to accurately assess sensitivity of potentiation to phosphorylation-level for G551D-CFTR, we repeated these experiments with prolonged activation in the presence of MgATP and PKA (i.e., up to 52 minutes) prior to addition of 0.2 µM VX-770. Representative current traces and summary data are shown in Fig. 7. Once G551D-CFTR was fully activated, potentiation by VX-770 exhibited phosphorylation-level dependence similar to WT-CFTR (Fig. 7A,B,E). G551A-CFTR also was potentiated by VX-770 in a manner sensitive to phosphorylation level when channels were fully activated (Fig. 7C–E). We note that when channels were fully activated by PKA, the response to VX-770 was greatly diminished. The magnitude of G551D-CFTR potentiation by VX-770 therefore depends on channel phosphorylation level, with the ultrahigh potentiation efficacy shown in Fig. 3 accounted for in part by incomplete channel pre-activation.

**Figure 8:**
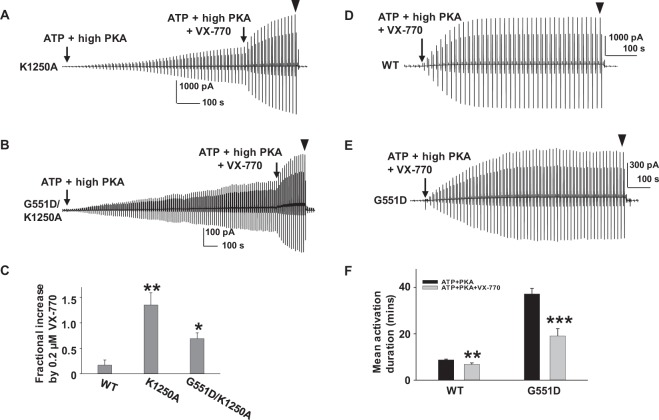
Cytoplasmic 0.2 µM VX-770 strongly potentiates K1250A- and G551D/K1250A-CFTR and promotes activation of WT- as well as G551D-CFTR. K1250A- (**A**) and G551D/K1250A-CFTR (**B**) were recorded under the same experimental conditions as in Fig. 2. Fractional increases for K1250A- and G551D/K1250A-CFTR were calculated with the equation described in Fig. 2. Summary data are shown in (**C**). *P < 0.05, and **P < 0.01 compared to WT-CFTR. *n* = 10 for K1250A; *n* = 6 for G551D/K1250A. VX-770 accelerated activation of WT- as well as G551D-CFTR. WT- (**D**) and G551D-CFTR (**E**) were activated in the presence of 0.2 µM VX-770 + 1 mM MgATP + 127.6 U/ml PKA. ▼, 10 µM CFTR_inh_172. (**F**) Summary data for mean activation durations. **P < 0.01 and *** *P* < 0.001 compared to control conditions (ATP + PKA). *n* = 9 for WT. *n* = 12 for G551D.

Mutation K1250A was proposed to abolish CFTR ATPase activity, although controversy exists regarding effect of this mutation on open probability, with reports ranging from half that of WT-CFTR to nearly 1 in the presence of 1 mM MgATP^40–43^. Based on our findings, pharmacological modulators such as VX-770 may be unable to potentiate K1250A-CFTR once it has been maximally activated (i.e., open probability close to 1). This phenomenon has been referred to as the “ceiling effect” in pharmacological terms^44^. We examined potentiation of K1250A- and G551D/K1250A-CFTR by VX-770; results are shown in Fig. 8. Once K1250A-CFTR channels were fully activated with high PKA and 1 mM MgATP, 0.2 µM VX-770 increased macroscopic current (Fig. 8A,C), with potentiation significantly higher than that of WT-CFTR. The data support previous findings indicating that open probability of K1250A-CFTR in 1 mM MgATP + 127.6 U/ml PKA must be much lower than WT-CFTR rather than close to 1. Similar data were collected for G551D/K1250A-CFTR channels (Fig. 8B,C). In summary, our data indicating substantial potentiation by VX-770 suggest that both K1250A- and G551D/K1250A-CFTR exhibit significant gating defects.

### VX-770 potentiates G551D-CFTR by multiple mechanisms

We previously reported that VX-770 reduced the closing rate of WT-CFTR^14^ and have now presented data showing that VX-770 potentiates CFTR in a phosphorylation-level dependent manner. These observations led us to hypothesize that VX-770 potentiates CFTR in multiple ways, and we tested this hypothesis as follows. First, we asked whether VX-770 facilitated activation of CFTR. As shown in Fig. 8D–F, we added 0.2 µM VX-770 along with 1 mM MgATP and 127.6 U/ml PKA as soon as the patch was pulled from oocytes. WT-CFTR activation was accelerated and estimated mean activation duration significantly shortened by VX-770. When the same experiment was performed with G551D-CFTR (Fig. 8E), activation of G551D-CFTR was again distinctly more rapid, with estimated mean activation duration reduced nearly 50% compared to absence of VX-770 (Fig. 8F). Hence, VX-770 may facilitate activation of G551D-CFTR channels.

**Figure 9:**
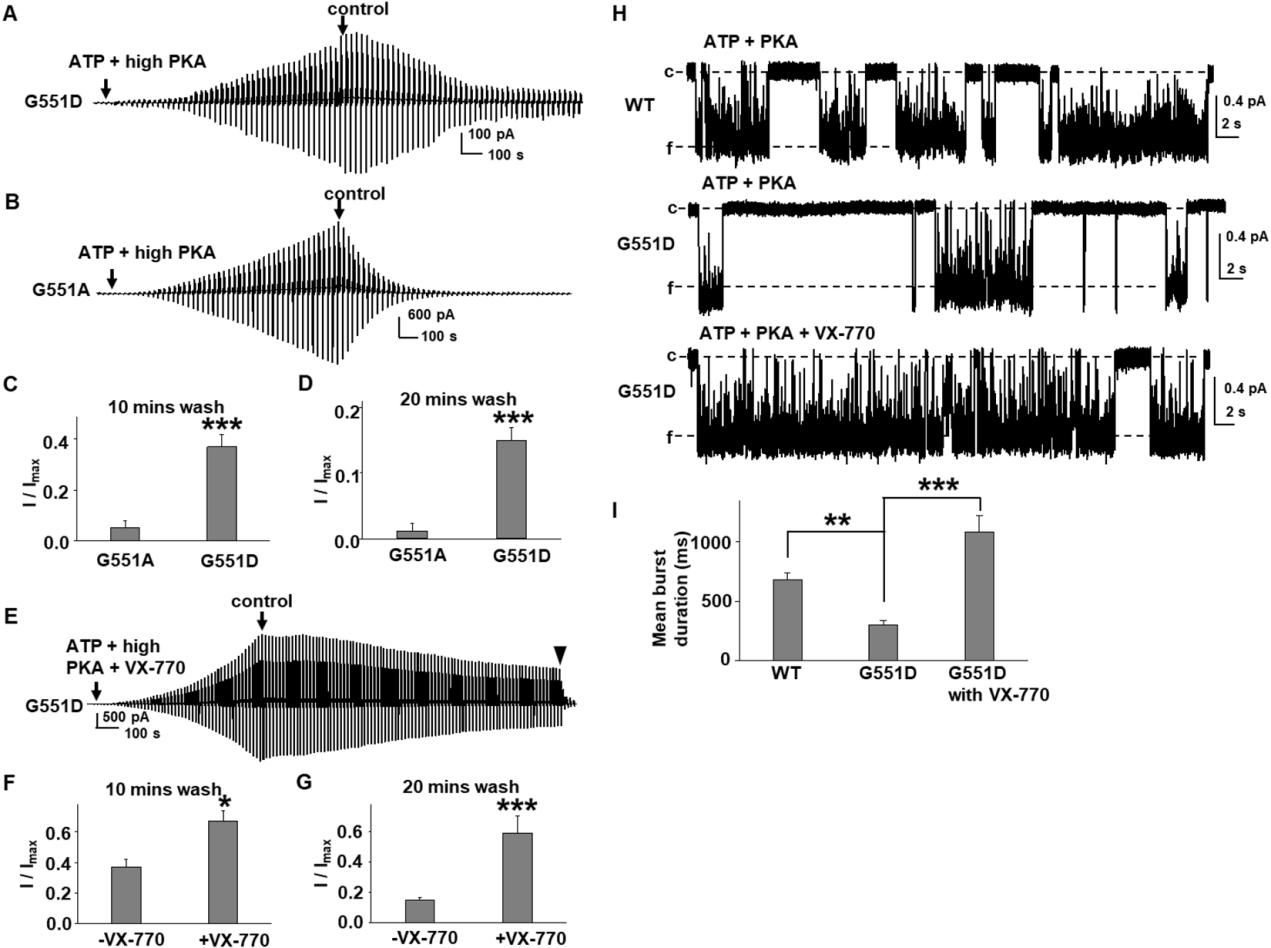
G551D-CFTR current exhibits decreased rate of deactivation upon removal of ATP and PKA. Representative current traces for G551D- (**A**) and G551A-CFTR (**B**) activated with 1 mM MgATP + 127.6 U/ml PKA then deactivated by washout of ATP and PKA with intracellular solution (control). A voltage ramp protocol described in the Methods section was applied every 5 s. Summary data for deactivation are shown in (**C**,**D**) and follow the equation (I/I_max = _I _(remaining current)_/I _(maximum current in ATP+PKA)_). ***, *P* < 0.001 compared with G551A. *n* = 5 for both G551A and G551D. (**E**) VX-770 significantly slowed the rate of deactivation of G551D-CFTR upon removal of ATP + PKA + VX-770. Representative current traces for G551D-CFTR activated with 1 mM MgATP + 127.6 U/ml PKA + 0.2 µM VX-770 for about 20 minutes then deactivated by washout with intracellular solution (control) for >25 minutes followed with 10 µM CFTR_inh_172 (▼). Summary data for deactivation are shown in (**F**,**G**). *P < 0.05 and ***P < 0.001 compared with G551D in the absence of VX-770. *n* = 5. (**H**) Representative single channel current traces of WT- and G551D-CFTR recorded in symmetrical 150 mM Cl^−^ solution. Data for G551D-CFTR is from the same patch for pre- and post-VX-770. Summary data for mean burst durations are shown in (**I**). Mean burst duration for G551D-CFTR was significantly shorter than for WT-CFTR. Mean burst duration for G551D-CFTR in the presence of VX-770 was significantly longer than for ATP + PKA alone. *n* = 5 for G551D-CFTR. WT-CFTR data cited from previous publication^14^. **P < 0.01 compared with WT-CFTR. ***P < 0.001 compared with G551D-CFTR in pre-VX-770 condition.

Next, we determined whether VX-770 reduced closing rate of G551D-CFTR following washout of ATP (as was observed for WT-CFTR)^14^. G551D-CFTR was activated with 1 mM MgATP + 127.6 U/ml PKA and the patch moved to a fast perfusion system by which channels were deactivated with control solution (no ATP or PKA). A representative current trace is shown in Fig. 9A. In these experiments, G551D-CFTR channels were only partially activated due to brief exposure to PKA. As noted previously, G551D-CFTR was slow to fully deactivate such that more than 15% current was remaining even after 20 minutes exposure to control solution^37^. Summary data (Fig. 9C–D) indicate that deactivation is significantly slower in G551D-CFTR than in G551A-CFTR, strongly supporting an electrostatic interaction between D551 and K1250 that would be absent in G551A-CFTR. Indeed, deactivation of G551A-CFTR was significantly faster than that of G551D-CFTR (Fig. 9B,C); G551A-CFTR channels exhibited near complete inactivation after 10 minutes wash-out with control solution. These data, combined with results in Fig. 6, suggest that an electrostatic interaction between D551 and K1250 is involved in both slow activation and slow deactivation demonstrated here for G551D-CFTR. Following exposure to VX-770, the deactivation of G551D-CFTR was significantly slower compared to findings in absence of VX-770 (Fig. 9E–G).

Lastly, we asked whether VX-770 modulated the single channel behavior of G551D-CFTR. We performed excised inside-out single channel recordings of WT- and G551D-CFTR at Vm = −100 mV in the presence of 1 mM MgATP and 127.6 U/ml PKA. Representative single channel current traces of WT- and G551D-CFTR are shown in Fig. 9H. We note that because G551D-CFTR activation duration is extremely long (see above), we allowed G551D-CFTR to be activated by MgATP and PKA for only 10 minutes then exposed the patches to 0.2 µM VX-770 in the presence of ATP and PKA, in order to maintain high quality seal resistances needed for these recordings. The experimental protocol was otherwise similar to that used to generate the macroscopic recordings shown in Fig. 3F. We analyzed the mean burst duration of G551D-CFTR without and with VX-770; summary data are shown in Fig. 9I. Under these conditions, mean burst duration of G551D-CFTR is significantly shorter than that of WT-CFTR^34,35^. VX-770 significantly prolonged mean burst duration of G551D-CFTR compared to with ATP and PKA alone, consistent with the notion that VX-770 may stabilize the G551D open state. In conclusion, mutation of G to D at position 551 in CFTR impacts both activation and deactivation, and VX-770 impacts both activation and deactivation processes conferred by G551D-CFTR.

In summary, VX-770 potentiated several CFTR disease-associated mutants expressed in oocytes, in a manner dependent upon phosphorylation level regardless of mutation location across the whole CFTR protein. Not only in WT, but also in individual mutations, VX-770 modulated CFTR functions through multiple mechanisms^14,16^. For example, VX-770 accelerated activation of G551D-CFTR possibly by increasing both total number of activated channels and the open probability and slowed deactivation of G551D-CFTR by stabilizing its open state. The complicated modulation of G551D-CFTR channels by VX-770 could contribute to the ultrahigh efficacy on G551D-CFTR. Taken together, these data strongly suggest that VX-770 possibly binds in more than one binding pocket in WT- as well as mutant CFTR in order to allosterically modulate the channel protein.

### Potentiation of CFTR in FRT cells also depends on activity level

**Figure 10:**
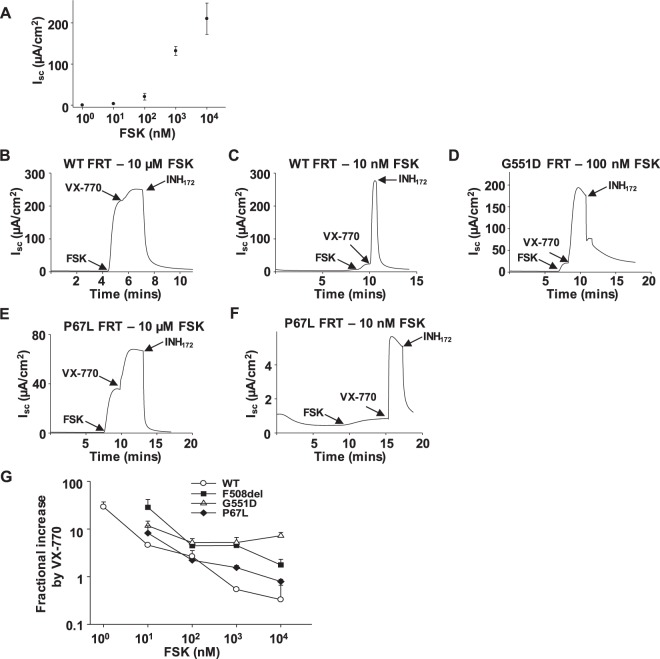
Efficacy of potentiation by 1 µM VX-770 of WT-, F508del-, P67L-, and G551D-CFTR expressed in FRT cells by 1 µM VX-770 is inversely proportional to the phosphorylation level. (**A**) Summary data showing a dose-response curve for forskolin (FSK) in FRT cells expressing WT-CFTR. *n* = 4. (**B**,**C**) Representative current traces of WT-CFTR activated by high and low FSK. Activation of FRT cells expressing WT-CFTR using 10 µM FSK led to near maximal currents; subsequent treatment with VX-770 led to a potentiation ratio of 0.5 ± 0.03 (Fractional increase = I_post_/I_pre_ − 1; I_post_, with VX-770; I_pre_, without VX-770). Channels activated with 10 nM FSK were potentiated by 4.6 ± 0.15 fold. Representative current traces of G551D- (**D**) and P67L-CFTR (**E**,**F**) are shown. INH_172_, 10 µM CFTR_inh_172. (**G**) VX-770 potentiated WT-, as well as three CFTR variants in a manner variably dependent upon FSK concentration. WT-CFTR: *P* < 0.05, comparing 10 µM FSK *vs* 10 nM FSK; *P* < 0.05, 10 µM *vs* 100 nM. G551D-CFTR: *P* < 0.05, 10 µM vs 10 nM. F508del-CFTR: *P* < 0.05, 10 µM *vs* 10 nM. P67LCFTR: *P* < 0.05, 10 µM *vs* 10 nM. *n* = 5 for WT-CFTR. *n* = 3 each for F508del-, G551D-, and P67L-CFTR.

Our data thus far suggest that the potentiation of CFTR by VX-770 is mediated by phosphorylation level when the channel is expressed heterologously in *Xenopus* oocytes. We next asked whether the activation level of CFTR affected potentiation efficacy in a more physiologically relevant system, FRT epithelial cells. The FRT cell line was chosen because it is commonly used to screen for new CFTR potentiators in industry and academic laboratories. Figure 10A shows graded activation of WT-CFTR channels by FSK and reaffirms the conclusion that it is an inherent characteristic of CFTR. When WT-CFTR was fully activated by 10 µM FSK, subsequent exposure to 1 µM VX-770 led to a 0.32 ± 0.33 fold increase in transepithelial current (I_sc_), shown to be attributable to CFTR by its sensitivity to the CFTR inhibitor INH_172_ (Fig. 10B). However, at 10 nM forskolin, 1 µM VX-770 potentiated WT-CFTR by approximately 5-fold (Fig. 10C). This inverse relationship was observed over the entire 10^5^-fold range of FSK concentrations tested (Fig. 10G). We next tested three CFTR variants bearing the most commonly occurring mutation, F508del, P67L, and G551D^13^ (Fig. 10D–G). The concentration of FSK used to activate CFTR currents influenced the level of potentiation of mutants by VX-770^45^, although the impact of phosphorylation level was much less for G551D-CFTR than for the other three variants. Importantly, when 10 nM FSK was used, VX-770 potentiated P67L- and F508del-CFTR to a similar level as G551D-CFTR (about 10 fold). This suggests that with submaximal activation of P67L-CFTR, VX-770 is efficacious enough to benefit patients bearing these mutations if channels are properly trafficked to the plasma membrane.

### Potentiation of WT-CFTR in HBE cells also depends on activity level

All systems tested to this point involved heterologously expressed CFTR, so we next asked whether this relationship occurred in primary HBE cells that endogenously express WT-CFTR. After blocking epithelial sodium channel (ENaC) currents with amiloride (Amil), we activated CFTR using either 10 nM or 10 µM FSK (Supplementary Fig. 2A,B). As in the FRT cells, CFTR current amplitude was proportional to the concentration of FSK used to activate the channels. Additionally, the magnitude of potentiation by 1 µM VX-770 was inversely proportional to the FSK concentration. In the presence of 10 nM FSK, VX-770 led to a 5.7-fold potentiation of current whereas the potentiation ratio in the presence of 10 µM FSK was 0.18 (Supplementary Fig. 2C). These data strongly suggest that the VX-770-mediated potentiation of CFTR in primary bronchial epithelial cells is dependent upon phosphorylation level, similar to that observed in the heterologous expression systems.

## Discussion

In these studies, we investigated the dependence of VX-770-mediated potentiation on channel phosphorylation level in WT-CFTR as well as six disease-associated CFTR mutants. We found: (1) VX-770 potentiates WT-, E193K-, K1060T-, and N1303K-CFTR in a manner dependent upon the level of PKA-mediated phosphorylation; (2) VX-770 potentiates P67L- and F508del-CFTR with less apparent phosphorylation-dependence under conditions of controlled PKA activity due to the alteration of PKA sensitivity in these mutants; (3) VX-770 potentiates G551D-CFTR in a phosphorylation-dependent manner even when the channels are fully activated, while the phosphorylation-dependence of potentiation is concealed when the G551D-CFTR channels are only partially activated; (4) VX-770-mediated potentiation of G551D-CFTR involves multiple mechanisms, including acceleration of activation and deceleration of deactivation; (5) Potentiation by VX-770 of WT-, P67L-, F508del-, and G551D-CFTR exhibits phosphorylation-dependence in FRT cells; (6) Potentiation by VX-770 of WT-CFTR exhibits phosphorylation-dependence in primary bronchial epithelial cells; and (7) Not only VX-770, but potentiator P2 also exhibits efficacy that is sensitive to phosphorylation level.

VX-770 modulated CFTR function in multiple ways in an ATP-dependent manner and not an ATP-independent manner^14,16,18,46,47^. Although we did not study VX-770 potentiation modified by ATP-dependent gating here, other groups have proposed that VX-770 potentiation of CFTR is tightly related to ATP-dependent gating^48,49^. We summarize our findings as follows: (1) Pre-treatment of WT-CFTR with VX-770 significantly slowed channel deactivation upon removal of ATP^14^. (2) VX-770 promoted the activation of WT- and G551D-CFTR when CFTR channels were exposed to ATP + PKA + VX-770 simultaneously. (3) Similar to previous findings from Van Goor and colleagues, we found here that VX-770 potentiated multiple CFTR variants bearing mutations that are located throughout the CFTR protein^13,24^. (4) In addition, we found that VX-770 potentiated WT and mutant CFTR in a phosphorylation-level mediated manner regardless of the mutations’ locations. Furthermore, we point out our data here might not exclude the possibility that high levels of phosphorylation on the R domain by high PKA could reinforce ATP-dependent channel gating of CFTR. Consequently, the ATP-dependent gating enhancement might be related to less potentiation by VX-770 in high PKA condition^48^. More studies are needed to completely understand the possible interaction between ATP-dependent and phosphorylation-level mediated potentiation of CFTR by VX-770.

Gating of the CFTR channel is controlled by protein kinase-mediated phosphorylation of the R domain and binding and hydrolysis of ATP at the NBDs. It was commonly believed that the R domain simply moves out of the way after phosphorylation to allow the NBDs to form a dimer and the CFTR channel to open. One piece of evidence supporting this idea is that deleting the R domain from CFTR allows it to open in a manner independent of phosphorylation while still requiring MgATP^50^. However, the open probability and macroscopic current density of the R domain-deleted CFTR is only one third that of the WT channel, suggesting a fundamental difference between the two CFTR variants^50^. Another piece of evidence supporting the R domain contribution to channel gating of CFTR was proposed by Liu and colleagues recently, where they found that the R domain directly modulated CFTR channel gating from dephosphorylated (D) to phosphorylated (P) and maximally activated (M) states^5^. In addition, more and more evidence collected in the last decade supports the hypothesis that the phosphorylated R domain interacts directly with several intracellular parts of CFTR including the N-terminus, ICLs, NBDs, and the C-terminus, thus affecting channel activity^7,8,51–54^. We found that the disease mutations P67L and N66S in the Lasso motif significantly lengthened activation duration and shifted PKA sensitivity (Fig. 5), suggesting that the Lasso motif might have a direct contact with the R domain. The Lasso motif may be similar to extracellular loop 1 in that it requires a finely-tuned structure in order to precisely perform its function. Consequently, mutations like P67L and N66S could destabilize the loop structure and significantly shift interactions between the R domain and the Lasso motif, while a stable loop structure may be a prerequisite for R domain modulation of CFTR channel function^23^. Furthermore, the lengthened activation duration of P67L and N66S could also be related to altered kinetics and energetics of their activation modulated by the R domain’s phosphorylation condition^5^.

As demonstrated previously, F508del, the most common disease mutation in the American CF population, induces multiple defects including poor trafficking in mammalian cells, gating dysfunction, and fast endocytosis after the protein is trafficked to the plasma membrane^11^. The gating dysfunction of F508del-CFTR may result from destabilization of the interactions of ICL3 and 4 with NBD1, at the site of F508, and impairment of NBD dimerization^55,56^. In addition, the aromatic side chain of F508 was proposed to have a strong impact on CFTR function^33^. We found that similar to F508del-CFTR, variants bearing F508S and F508C demonstrated altered PKA sensitivity suggesting that phenylalanine in position 508 also might be necessary for maintaining local structure and interactions with the R domain. In summary, our data support the previous reports that the R domain interacts with multiple parts of CFTR in regulating CFTR channel function. Data presented here suggest that these graded interactions between the R domain and other parts of the channel, as regulated by the level of PKA-mediated phosphorylation, also determine the sensitivity to potentiation by VX-770 and P2 for all variants we studied here. Sensitivity to phosphorylation level was seen for potentiation of WT-, F508del-, G551D-, and P67L-CFTR in both oocytes and FRT cells, and for WT-CFTR in primary airway epithelial cells, albeit not to the same degree, likely due to differences in the means of channel activation by PKA (direct application vs. via forskolin). Hence, we conclude that phosphorylation-dependence of potentiation may be a fundamental characteristic of the allosteric regulation of CFTR function.

G551D is one of the CFTR mutations that has drawn the most attention in the last 20 years. Thus far, in comparison to WT-, G551D-CFTR has been demonstrated to have: (1) lower open probability and shorter open burst duration; (2) unique ATP-independent activity; (3) slow activation by PKA activators forskolin and IBMX; (4) slow deactivation in whole oocytes; (5) reduction in rate of ATP hydrolysis; and more^34,35,37,57,58^. Unfortunately, the mechanisms behind G551D-CFTR’s unusual behavior remain unclear. Our previous simulation results predicted that the D at position 551 may form an electrostatic interaction with K at position 1250 that both perturbs ATP binding and slows down activation of G551D-CFTR. The results shown here strongly support this prediction, including: (1) The estimated rate of PKA-mediated activation of macroscopic currents (from measurements of activation duration) is much slower for G551D- than WT-CFTR under the same experimental conditions; (2) These effects are lost in G551A- and G551D/K1250A-CFTR; (3) Unlike WT-CFTR which deactivates rapidly within only a couple of minutes after washout of ATP and PKA in control solution^14^, G551D-CFTR deactivation rates after removal of ATP and PKA are extremely low; (4) This effect is lost in G551A-CFTR. These results are also supported by previous observations^35,37^. While we cannot determine the rate of ATP (or ADP + P_i_) dissociation from the NBDs, G551D-CFTR clearly remains open for a long time in ATP–free solution. Taken together, we propose that the unique and complicated behavior of G551D-CFTR results from an electrostatic interaction between D at 551 and K at 1250. The efficacy of potentiation of G551D- and G551A-CFTR by VX-770 is similar to WT- when G551D- and G551A-CFTR channels were fully activated by direct application of PKA, and potentiation exhibits phosphorylation dependence under these conditions. But, the unique behavior of G551D-CFTR likely contributes to the vigorous potentiation by VX-770 observed at all phosphorylation levels in more physiological conditions, such as FRT cells (Fig. 10), since G551D-CFTR exhibits such low open probability^35^. This likely explains why potentiation of G551D-CFTR is uniquely less sensitive to phosphorylation level (Fig. 10G).

In this study we present evidence that the ability of VX-770 to increase CFTR activity is inversely related to the channel’s initial activity level before the drug is applied. This “ceiling effect” here in CFTR specifically reflects the physical limit for how much current a single gating CFTR channel can pass in time, which consequently limits the apparent ability of VX-770 to increase channel activity. This interaction between phosphorylation level and efficacy of VX-770-mediated potentiation is conserved across cells expressing WT- or mutant CFTR variants endogenously or heterologously. This finding has practical implications because phosphorylation levels that tune CFTR activity in bodily tissues likely are far below the maximum levels achieved with direct application of PKA, a notion supported by two observations. First, long-term PKA-mediated activation of CFTR by cholera toxin poisoning leads to levels of water loss that far exceed normal physiological levels^59^. Second, nasal potential difference (NPDs) in healthy individuals is increased by perfusion with isoproterenol due to an increment in CFTR activity from resting condition^60^. Hence, CFTR is not fully activated under normal physiological states. Consequently, our data suggest that screening drugs against submaximally activated CFTR is likely more physiologically relevant than screening against CFTR channels activated by high concentrations of PKA activators like forskolin and IBMX, or PKA, itself. Additionally, our data using P67L-FRT cells suggest that using a lower concentration of forskolin might enable the discovery of more potentiators by increasing the available dynamic range, thus avoiding the ceiling effect. Since there are not enough patients with the P67L or other ultra-orphan mutation genotypes to run a full-scale clinical trial, *in vitro* data will be a large consideration in determining whether VX-770 or other potentiators receive FDA approval^21^. Our results suggest that such drug screening should be performed on CFTR channels at a lower activation state.

Chronic VX-770 incubation has been reported to destabilize F508del-, not WT-, CFTR in the plasma membrane^61–63^. We have observed a shift in the concentration-dependent activation of WT-CFTR in FRT cells by FSK after 24 hour pre-incubation of 1 µM VX-770 (Supplementary Fig. 3). The half-maximal activation concentration of FSK (EC_50_) was significantly reduced with VX-770 chronic treatment while the maximal I_sc_ remained unaffected. These results indicate that WT-CFTR channels were activated more easily after VX-770 pre-incubation compared to the control group (DMSO). In fact, VX-770 might facilitate activation of CFTR channels both acutely and chronically (Fig. 8D–F; Supplementary Fig. 3). However, it is unknown whether VX-770 would affect phosphorylation levels of CFTR channels when it is used chronically. In addition, while this manuscript was under revision there were two publications that reported a possible binding pocket in the TMs for VX-770^64,65^. There is no doubt that more studies are required to fully understand the mechanism and binding sites of VX-770 in potentiation of CFTR to benefit the CF patient community.

## Supplementary information

Supplementary figure and legend
