# Supplementary material for: VX-770-mediated potentiation of numerous human CFTR disease mutants is influenced by phosphorylation level
Source: Sci Rep. 2019 Sep 17;9:13460. doi: 10.1038/s41598-019-49921-4 (PMC6749054; doi:10.1038/s41598-019-49921-4)
Supplement: Supplementary file 1 — Supplementary figure and legend [file 41598_2019_49921_MOESM1_ESM.pdf]

VX-770-mediated potentiation of numerous human CFTR disease mutants is influenced by phosphorylation level

Guiying Cui, Brandon B. Stauffer, Barry R. Imhoff, Andras Rab, Jeong Hong, Eric J. Sorscher, and Nael A. McCarty*

Division of Pulmonology, Allergy/Immunology, Cystic Fibrosis, and Sleep, Department of Pediatrics, Emory + Children's Center for Cystic Fibrosis and Airways Disease Research, Emory University School of Medicine and Children's Healthcare of Atlanta, 2015 Uppergate Drive, Atlanta, GA 30322

*Correspondence and requests for material should be addressed to N.A.M.

Nael A. McCarty, PhD
Division of Pulmonology, Allergy/Immunology, Cystic Fibrosis, and Sleep,
Department of Pediatrics,
Emory + Children's Center for Cystic Fibrosis and Airways Disease Research,
Emory University School of Medicine and Children's Healthcare of Atlanta,
2015 Uppergate Drive, Atlanta, GA 30322;
Phone No.: 404-727-3654;
Fax No.: 404-712-0920;
Email: namccar@emory.edu

Supplementary figure

Supplementary Fig. 1. Cytoplasmic 5 µM P2 potentiated WT-, E193K-, K1060T-, and N1303K-CFTR in a manner dependent upon phosphorylation level. Representative macropatch currents (WT: **A**, **B**; E193K: **C**, **D**; K1060T: **E**, **F**; N1303K: **G**, **H**) recorded in inside-out mode with symmetrical 150 mM Cl^- solution under the following experimental conditions: channels were activated in 1 mM MgATP + 6.4 U/ml or 127.6 U/ml PKA (low and high PKA, respectively) for ten minutes followed with addition of 5 µM P2 in the continuing presence of MgATP + PKA for about three minutes, and currents then were blocked by 10 µM $CFTR_{inh}172$ (▼). A voltage-ramp protocol described in the Methods section was applied every 5 s. **I**. Summary data for fractional increase of WT- and three disease mutants by P2 under each set of conditions (Fractional increase = $I_{(ATP+PKA+VX\text{-}770)} / I_{(ATP+PKA)} - 1$). WT: $n = 5$ for low PKA (high PKA data adopted from previous publication[1]). E193K: $n = 4$ for both high and low PKA. K1060T: $n = 6$ for high PKA; $n = 4$ for low PKA. N1303K: $n = 6$ for high PKA; $n = 5$ for low PKA. ***, $P < 0.001$ compared to high PKA condition.

Supplementary Fig. 2. Potentiation of endogenously expressed WT-CFTR by VX-770 is inversely proportional to PKA activity. **A**, **B**, When non-CF HBE cells expressing WT-CFTR were activated using 10 µM FSK, the VX-770 potentiation ratio was 0.18 ± 0.50 but increased to 5.5 ± 0.47 when 10 nM FSK was used. **C**. Summary data. *, $P < 0.05$ compared to 10 nM FSK. $n = 3$ for each.

Supplementary Fig. 3. Chronic VX-770 treatment reduced the half-maximal activation concentration (EC_{50}) of forskolin in activation of WT-CFTR. Representative I_{sc} traces of WT FRT cells recorded in Ussing chamber (A). WT FRT cells were pre-treated with 1 µM VX-770 (+VX-770) or the same volume of vehicle (DMSO, -VX-770) for 24 hours. Representative normalized currents (B) were fitted with the one site ligand binding equation with Sigmaplot 10.3. I_{max}: maximum current activated by FSK. Summary data for EC_{50} of FSK are shown in C. ***, $P < 0.001$ compared with control condition. D. Maximum ΔI_{sc} response to FSK observed in both groups. n=7 for each group.

Sup. Fig. 1

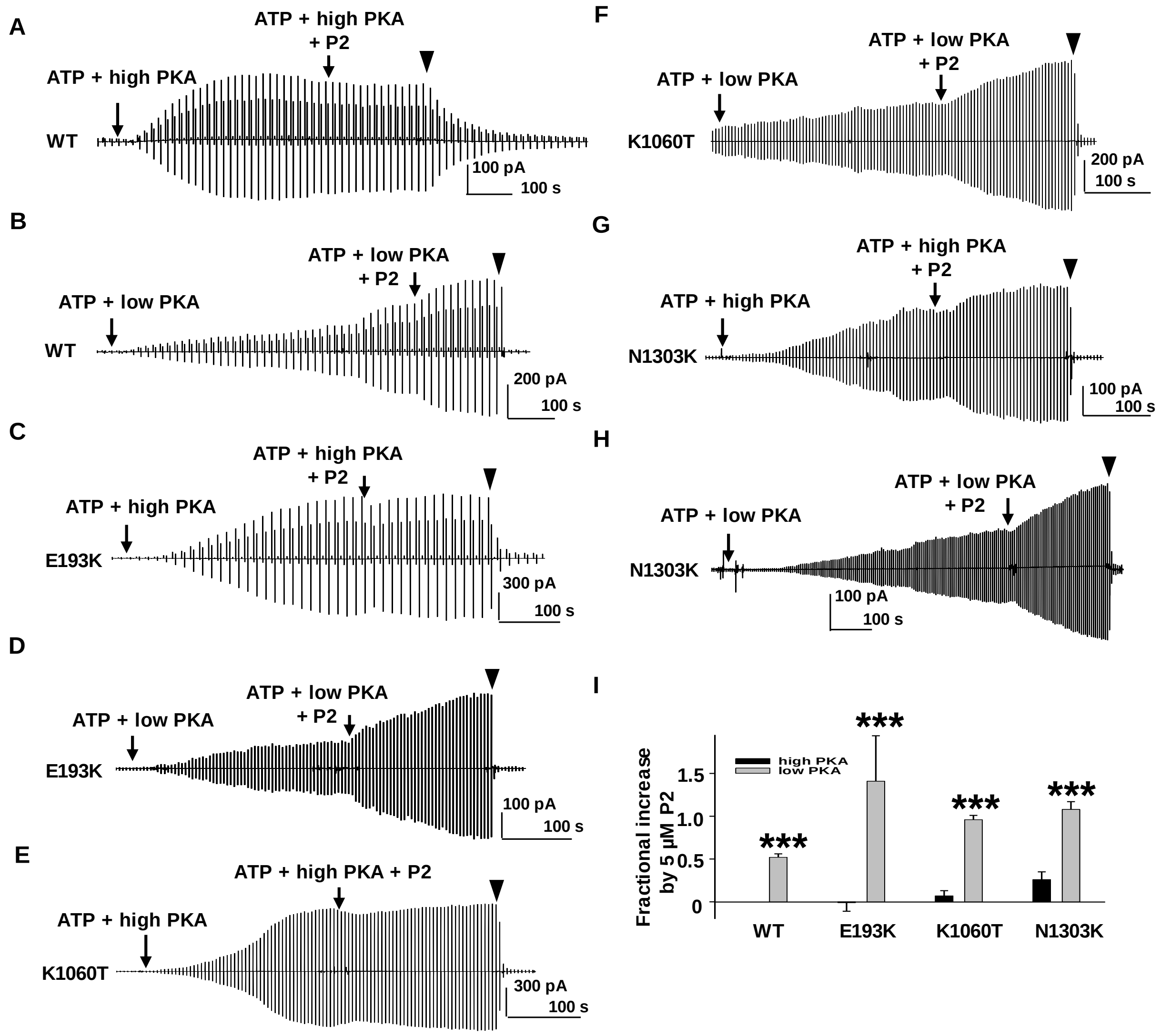

A
ATP + high PKA
ATP + high PKA
+ P2
WT
100 pA
100 s
B
ATP + low PKA
ATP + low PKA
+ P2
WT
200 pA
100 s
C
ATP + high PKA
ATP + high PKA
+ P2
E193K
300 pA
100 s
D
ATP + low PKA
ATP + low PKA
+ P2
E193K
100 pA
100 s
E
ATP + high PKA
ATP + high PKA + P2
K1060T
300 pA
100 s
F
ATP + low PKA
ATP + low PKA
+ P2
K1060T
200 pA
100 s
G
ATP + high PKA
ATP + high PKA
+ P2
N1303K
100 pA
100 s
H
ATP + low PKA
ATP + low PKA
+ P2
N1303K
100 pA
100 s
I
Fractional increase by 5 μM P2
high PKA
low PKA
***
1.5
1.0
0.5
0
WT
E193K
K1060T
N1303K

Sup. Fig. 2

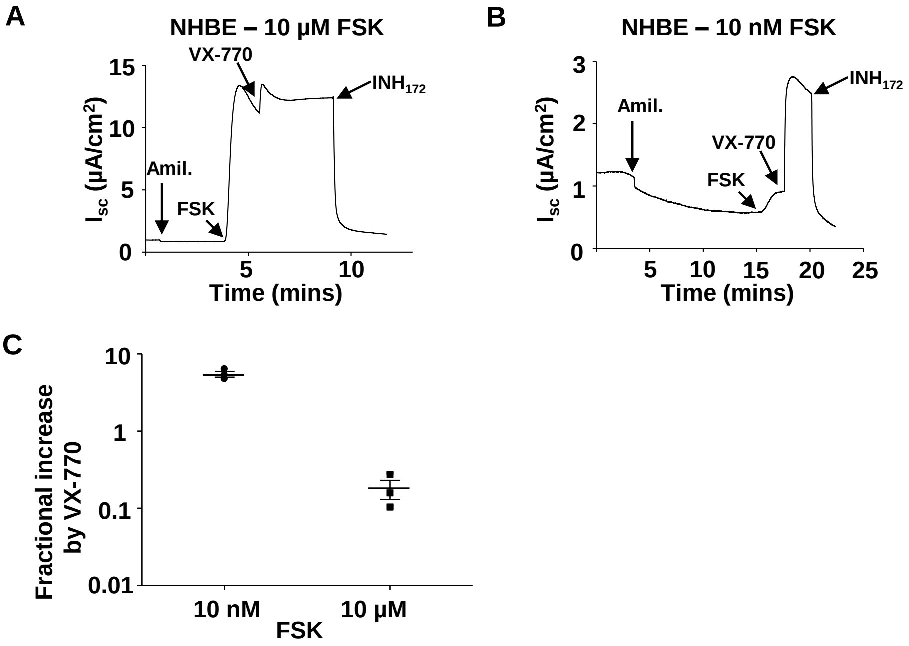
A
NHBE – 10 µM FSK
VX-770
INH172
Amil.
FSK
Isc (µA/cm2)
Time (mins)
B
NHBE – 10 nM FSK
Amil.
VX-770
FSK
INH172
Isc (µA/cm2)
Time (mins)
C
Fractional increase by VX-770
10 nM
10 µM
FSK

Sup. Fig. 3

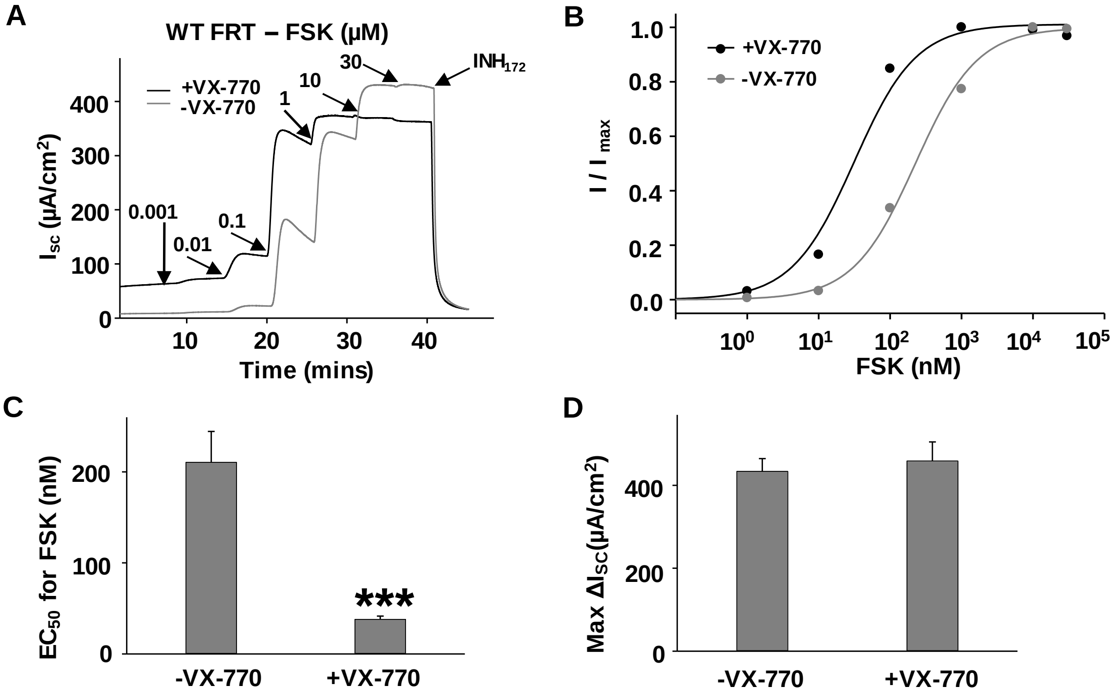

1. Cui G*, et al.* Potentiators exert distinct effects on human, murine, and Xenopus CFTR. *American journal of physiology Lung cellular and molecular physiology* **311**, L192-207 (2016).
